# [Corrigendum] Triptolide inhibits JAK2/STAT3 signaling and induces lethal autophagy through ROS generation in cisplatin-resistant SKOV3/DDP ovarian cancer cells

**DOI:** 10.3892/or.2026.9048

**Published:** 2026-01-13

**Authors:** Yanying Zhong, Fuyin Le, Jiao Cheng, Chen Luo, Xiali Zhang, Xingwu Wu, Fang Xu, Qi Zuo, Buzhen Tan

Oncol Rep 45: 69, 2021; DOI: 10.3892/or.2021.8020

Following the publication of the above article, the authors contacted the Editorial Office to explain that they had made inadvertent errors in compiling a couple of the figures in the above paper; first, regarding the immunohistochemical images shown in Fig. 2D on p. 5, the data panel shown correctly for the ‘LC3/TPL+DDP’ experiment contained an overlapping section with the ‘LC3/TPL’ data panel in the same figure part (the latter of which had been incorporated into this figure incorrectly). Secondly, the β-actin bands correctly shown in Fig. 3D on p. 6 had incorrectly been included to represent the JAK2 western blot data in Fig. 4F on p. 7.

However, the authors were able to re-examine their original data, and realized how these errors had occurred. The revised and corrected versions of Figs. 2 and 4, now showing the correct data for the ‘LC3/TPL’ experiment in Fig. 2D and the JAK2 western blot data in Fig. 4F, are shown on the next two pages. Note that the errors made with the assembly of the data in these figures did not affect the overall conclusions reported in the paper. The authors apologize to the Editor of *Oncology Reports* and to the readership for any inconvenience caused.

## Figures and Tables

**Figure 2.**
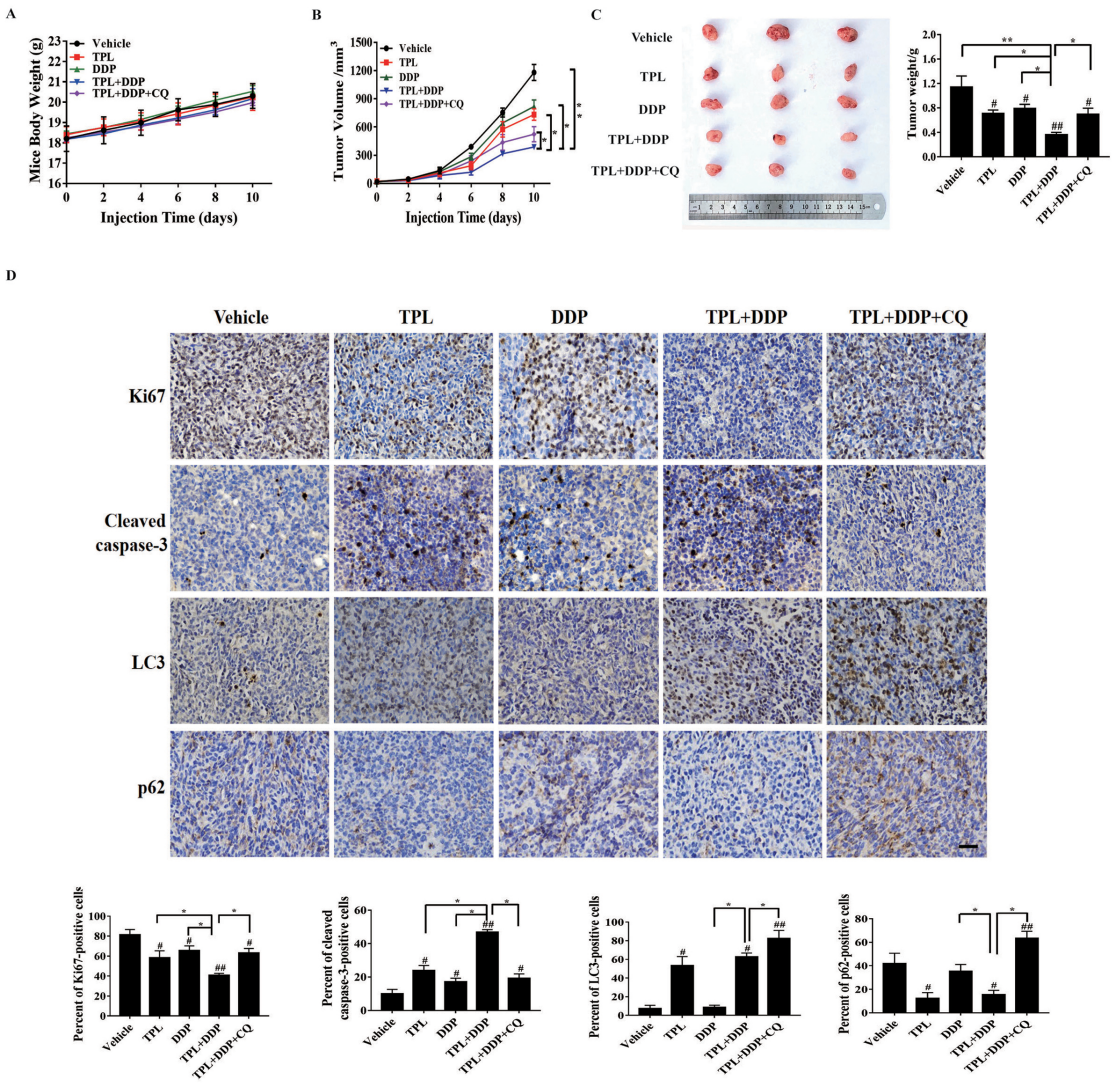
TPL potentiates the antitumor effect of DDP through autophagy induction *in vivo.* Mice bearing SKOV3/DDP tumor cells were intraperitoneally injected with PBS, TPL, DDP, TPL + DDP, and TPL + DDP + CQ. (A) The body weight of mice and (B) tumor volume were measured every two days during the administration period. At the end of experiment, tumors were removed, photographed and (C) weighed. (D) Immunohistochemical analysis (Ki67, cleaved caspase-3, LC3 and p62 staining) of tumor tissue sections isolated from the indicated groups of mice (magnification, ×400). Scale bar, 100 µm. *P<0.05 and **P<0.01 vs. the TPL + DDP group. ^#^P<0.05 and ^##^P<0.01 vs. the Vehicle group. TPL, triptolide; DDP, cisplatin; CQ, chloroquine; Con, control.

**Figure 4.**
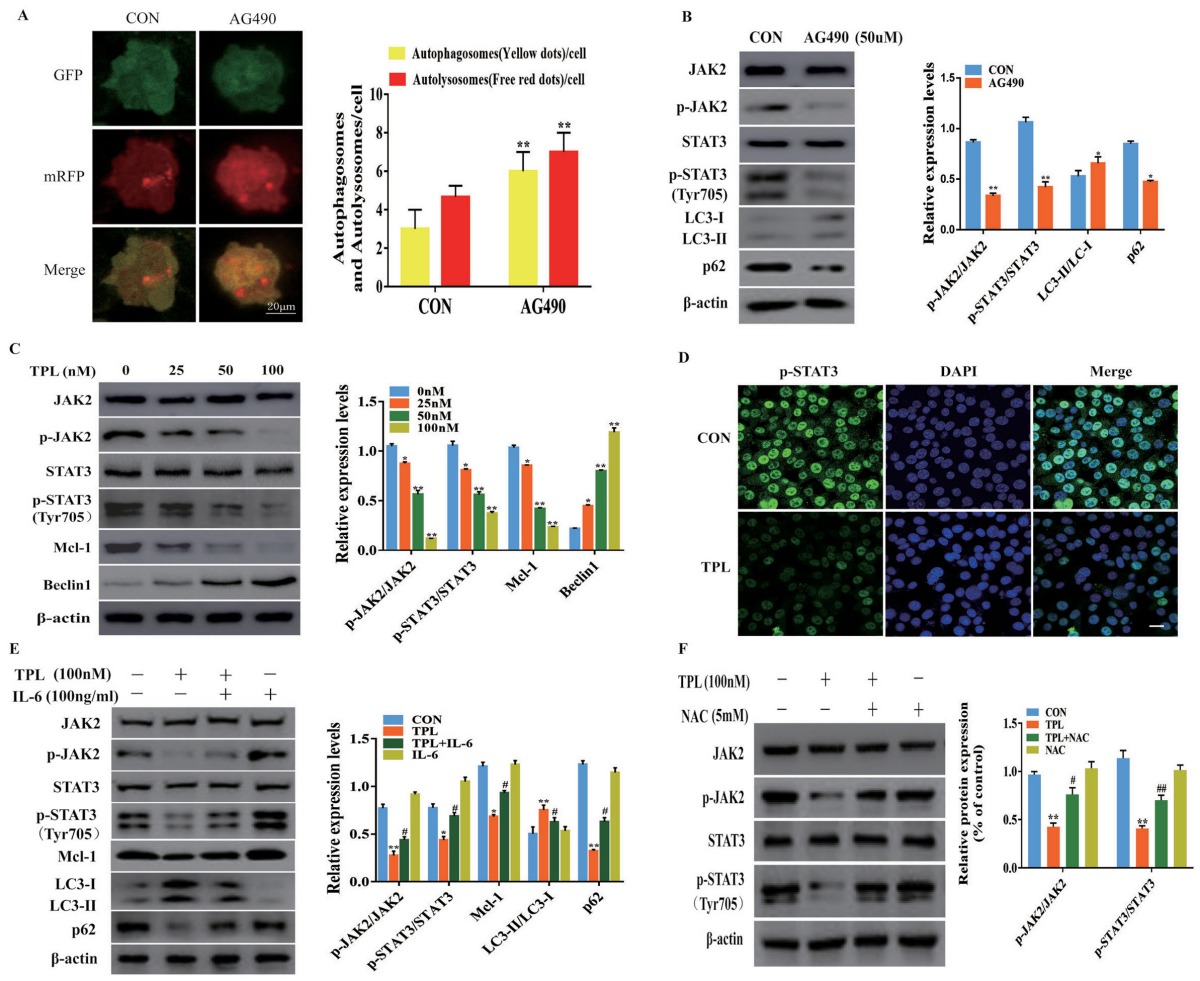
ROS generation is upstream of JAK2/STAT3 inactivation in TPL-induced autophagy. (A and B) SKOV3/DDP cells were treated with or without 50 µM AG490 for 24 h, and (A) the autophagosomes (yellow puncta) and the autolysosomes (red puncta) were detected using a fluorescence microscope. Scale bar, 20 µm. (B) The expression levels of p-JAK2, p-STAT3 (Y705), LC3 and p62 were compared by western blot analysis. (C) Western blotting revealed the expression of JAK2, p-JAK2, STAT3, p-STAT3 (Y705), Mcl-1 and Beclin1 in SKOV3/DDP cells after treatment with variable doses of TPL for 24 h. (D) SKOV3/DDP cells were treated with or without 100 nM TPL for 24 h, then the expression of p-STAT3 (Y705) was examined using immunofluorescence staining (magnification, ×400). The cell nuclei were stained with DAPI (blue). Scale bar, 20 µm. (E) SKOV3/DDP cells were incubated without or with TPL (100 nM) in the presence or absence of IL-6 (100 ng/ml) for 24 h. Mcl-1, LC3 and p62 were detected by western blot analysis. (F) SKOV3/DDP cells were treated with NAC (5 mM) for 1 h prior to exposure to TPL (100 nM) for 24 h, and then the p-JAK2 and p-STAT3 (Y705) expression were evaluated with western blotting. *P<0.05 and **P<0.01 vs. the Con group. ^#^P<0.05 and ^##^P<0.01 vs. the TPL group. ROS, reactive oxygen species; JAK2, Janus kinase 2; STAT3, signal transducer and activator of transcription-3; TPL, triptolide; DDP, cisplatin; p-phosphorylated; Mcl-1, myeloid cell leukemia-1; IL, interleukin; NAC, N-acetyl-l-cysteine; Con, Control.

